# New Coordination Polymers with Unexpected Octahedral copper(II) Geometry: Synthesis, Supramolecular and Theoretical Study

**DOI:** 10.1021/acsomega.5c07364

**Published:** 2026-05-11

**Authors:** Yair Alvarez-Ricardo, Nicolás Puentes-Díaz, Mario A. Macías, Jorge Alí-Torres, John J. Hurtado

**Affiliations:** † Grupo de Investigación en Química Inorgánica, Catálisis y Bioinorgánica, Departamento de Química, 27991 Universidad de Los Andes, Carrera 1 No. 18A-12, 111711 Bogotá, Colombia; ‡ Departamento de Química, 28021 Universidad Nacional de Colombia—Sede Bogotá, 111321 Bogotá, Colombia; § Cristalografía y Química de Materiales, CrisQuimMat, Departamento de Química, Universidad de los Andes, 111711 Bogotá, Colombia

## Abstract

In this work, we report the synthesis of two new copper­(II) complexes that preferentially assemble into coordination polymers through potential N,N,N- and N,C,N-type pincer ligands, with the metal centers adopting octahedral geometries. The crystallographic analysis of coordination polymers 2,6-bis­((1H-1,2,4-triazol-1-yl)­methyl)­pyridine-CuCl_2_ (C_1_), 3,5-bis­(1,2,4-triazol-1-ylmethyl)­toluene-CuCl_2_ (C_2_), and 2,6-bis­((1*H*-1,2,4-triazol-1-yl)­methyl)­benzene-CuCl_2_ (C_3_) reveals polymeric structures formed through triazole ligands imposing *cis* conformations and generating comparable unit cells. While in the case of ligands it was observed that 2,6-bis­((1*H*-1,2,4-triazol-1-yl)­methyl)­pyridine (L_1_) forms a 3D hydrogen-bonding network via triazoles and pyridine nitrogen, 3,5-bis­(1,2,4-triazol-1-ylmethyl)­toluene (L_2_), lacking pyridine, adopts a simpler 1D chain stabilized by C–H···N and C–H···π interactions, with van der Waals forces between chains and with C2 symmetry and 2,6-bis­((1*H*-1,2,4-triazol-1-yl)­methyl)­benzene (L_3_) displays both *trans* and *cis* forms, creating layered structures and losing symmetry due to directional hydrogen bonds. The computational study established that the formation energy (Δ*E*_f_) of the polymer chains C_1_, C_2_, and C_3_ decreases linearly with chain growth, indicating progressive electronic stabilization, with C_2_ showing the most favorable trend (−2.97 kcal/mol per unit). Similarly, the average stabilization energy per monomer (Δ*E*_avg_) becomes progressively more negative, converging at a chain length of about ten units, with C_2_ again displaying the greatest stability (−2.68 kcal/mol per monomer). In addition, the crystal structure of two azole derivatives, L_2_ and L_3_, is presented.

## Graphical abstract

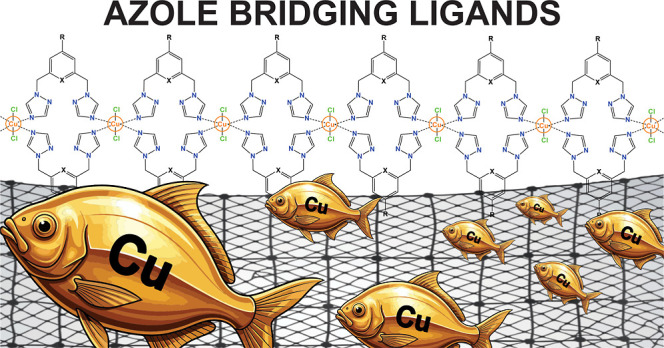

## 1 Introduction

Copper is a physiological metal that is abundant in the brain, where it binds to proteins such as amyloid-β (Aβ), a peptide involved in oxidative stress and found in senile plaques in Alzheimer’s patients.^1–3^ The copper­(II) ion of this metal coordinates to histidine, an amino acid released by mast cells during allergic reactions and essential for neuronal tissue maintenance and development. Histidine participates in the catalytic cycles of numerous enzymes through its imidazole side chain, whose basic nitrogen can capture hydrogen in physiological media.^4–6^

Azoles are known for their biological properties.^7–9^ They have emerged as promising anticancer agents because they possess unique structural features that make them particularly well-suited to interact with proteins and other molecules crucial for cell viability. Its electron-rich, five-membered imidazole ring (with two nitrogen donors) facilitates diverse noncovalent interactions, including hydrogen bonding, van der Waals contacts, and electrostatic forces, with molecular targets.^10,11^ This molecular interaction capacity is fundamental to their efficacy in cancer treatment, as it allows them to interfere with processes relevant to cell survival, such as DNA replication, thereby inhibiting cancer cell growth and proliferation.^12–14^

Due to the biological importance of copper and azoles, it is necessary to explore how these nitrogen-containing heterocyclic compounds could coordinate to this metal, that is, whether there is a preferential influence between the different nitrogen present in these 5-membered rings that favors one molecular geometry over another, and whether this preference is associated with an energetically favored product.^15^ Polymer complexes, in turn, represent a unique class of materials that combine the versatility of polymer backbones with the ability of metals to bind to ligands through coordination bonds. The study of polymeric structures is critical, as the spatial arrangement of ligands and metal ions directly affects both stability and reactivity. Unlike their low-molecular-weight analogues, these coordination polymers exhibit dynamic behaviors that enable tunable, multifunctional properties.^16–18^ A deeper understanding of their coordination modes, conformational flexibility, and local environments is therefore essential to unlock their full potential. Their hybrid nature enables properties such as electron transfer, selective catalysis, and sensing capabilities, yet many structure–function relationships remain unexplored. Advancing understanding in this field will enable rational design of smart materials for energy, biomedical, and nanotechnology applications.^19,20^

For this reason, we prepared azole derivatives with the expectation of obtaining Cu­(II) mononuclear complexes where the ligands exhibit a bidentate and tridentate coordination. However, we found that copper adopts an unexpected coordination geometry.

## 2 Results and Discussion

### 2.1 Synthesis and Characterization of Copper Complexes

The complexes (C_1_–C_3_) were prepared by dissolving the ligands (L_1_ – L_3_) and copper chloride (CuCl_2_) in methanol (MeOH) under constant agitation at room temperature (Scheme 1).

**Scheme 1:**
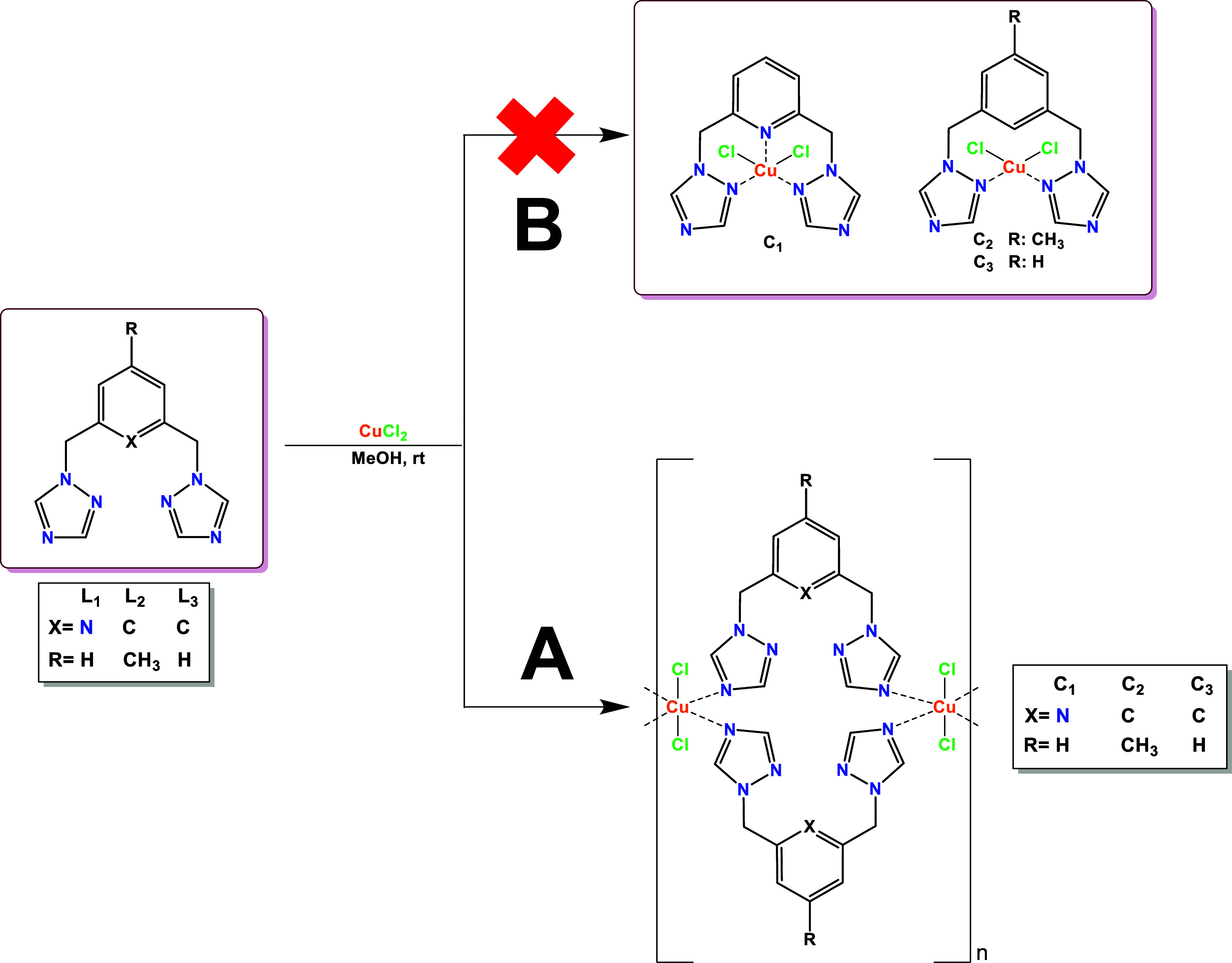
Synthesis of copper­(II) Complexes with L_1_, L_2,_ and L_3_: (A) Synthetic Route that Led to the Formation of the Fortuitous Coordination Polymers. (B) Expected Synthetic Route for Obtaining Mononuclear Cu­(II) Complexes Based on the Research Group’s Background

In a previous work from our group,^21^ complexes were synthesized from copper­(II) perchlorate hexahydrate and N,N,N-tridentate bis­(pyrazolylmethyl)­pyridine ligands, affording the expected tridentate complexes. As shown in Figure 1, the metal binds the pyrazole N2 atoms and the pyridine nitrogen.

**Figure 1:**
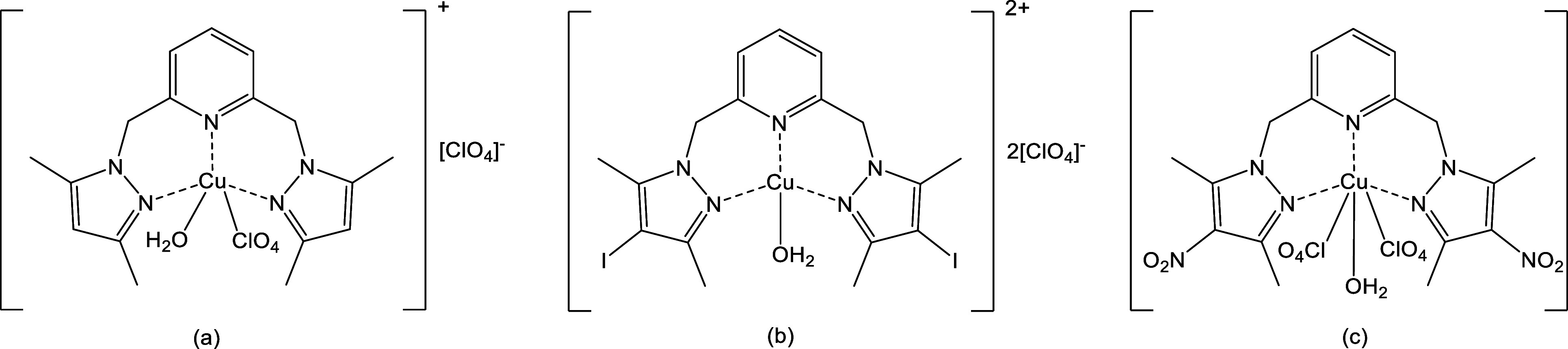
Copper­(II) complexes with tridentate bis­(pyrazolylmethyl) pyridine ligands.^21^

The above allowed us to establish the hypothesis that L_1_ would behave as a tridentate ligand, while L_2_ and L_3_ would behave as a bidentate species (Scheme 1B). However, we successfully determined the crystal structures of C_1_ and C_2_, whereas that of C_3_ was previously reported by Du et al.,^22^ showing the preference of the metal center to coordinate with the nitrogen of position 4 from the 1,2,4-triazole, which favors the octahedral geometry over the tetrahedral or trigonal bipyramid expected with mononuclear complexes. Due to the structural similarities between L_2_ and L_3_, the same geometry was observed for the metallic center in the C_2_ and C_3_ polymers.

Surprisingly, in C_1_, the Cu­(II) showed the same geometry, and it was found that, despite nitrogen in the pyridine ring of L_1_, the preferential coordination mode of the ligands in the polymers is directed by the nitrogen at position 4 from 1,2,4-triazole (Scheme 1A).

FTIR spectra confirm ligand–metal coordination through shifts in band positions, intensities, and shapes. The C=N stretching vibrations were observed at 1269 and 1261 cm^–1^ for L_1_ and L_2,_ respectively. However, the wavenumber shifts at 1280 cm^–1^ for C_1_ and C_2_.

Furthermore, for L_1_ and L_2,_ the N–N stretching vibration was observed at 1423 and 1431 cm^–1^, respectively. Upon coordination with the copper­(II) center, these bands are shifted to 1427 cm^–1^ in C_1_ and 1435 cm^–1^ in C_2_ (Figures S1–S4).^22,23^ The shifts to higher frequencies observed in the infrared spectrum then suggest modifications in the bond distances resulting when polymers are formed.

These findings enabled us to perform a supramolecular analysis to understand the interactions present in the solid state and, in parallel, a computational study to rationalize the structural preferences of these coordination polymers.

### 2.2 Crystallographic Analysis

Ligands L_1_, L_2_, and L_3_ were synthesized and characterized by single-crystal X-ray diffraction (SCXRD), with the structure of L_1_ having been reported previously.^24^ The ORTEP drawings of the molecular structures are shown in Figure 2. Table 1 summarizes the crystallographic data of the compounds L_2_ and L_3_.

**Figure 2:**
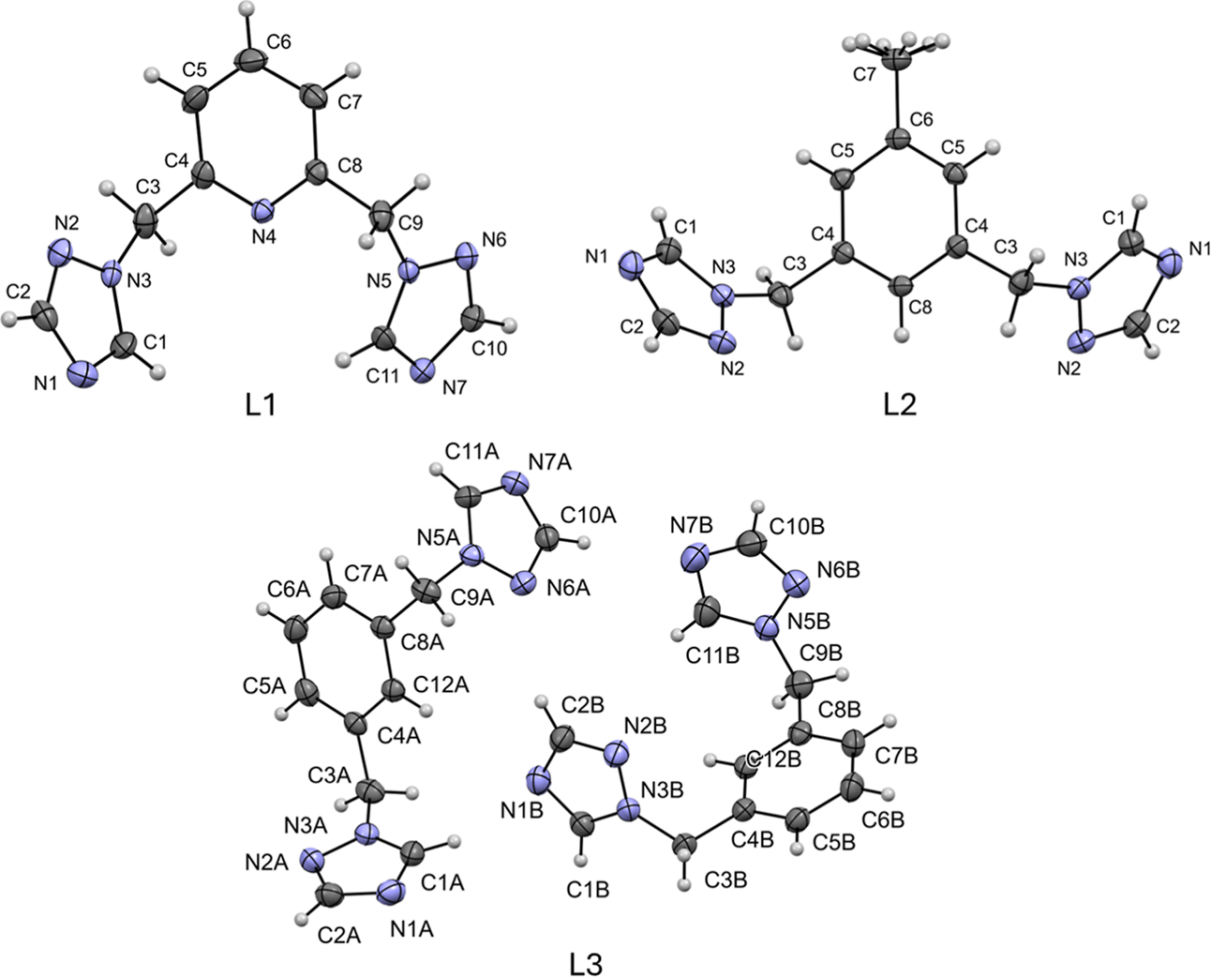
Molecular structures of compounds L_1_, L_2_, and L_3_ with anisotropic thermal vibration ellipsoids at the 30% probability level. The hydrogen atoms are shown as spheres of arbitrary radius.

**Table 1:** Crystallographic Data of Compounds L_2_, L_3_, C_1_, and C_2_

crystal data	L_2_	L_3_	C_1_	C_2_
CCDC	2473469	2473468	2473467	2473470
chemical formula	C_13_H_14_N_6_	C_12_H_12_N_6_	C_22_H_22_Cl_2_CuN_14_	C_26_H_28_ Cl_2_CuN_12_
*M*_r_	254.30	240.28	616.99	643.05
crystal system, space group	monoclinic, *I*2/*a*	monoclinic, *P*2_1_/*c*	triclinic, *P*1̅	triclinic, *P*1̅
*a* (Å)	7.0815 (4)	28.726 (3)	7.2273 (8)	7.9542 (11)
*b* (Å)	8.0958 (5)	4.4790 (4)	8.6294 (10)	8.7940 (14)
*c* (Å)	21.4696 (14)	19.3428 (19)	10.8808 (13)	10.8504 (16)
α (°)	90.0	90.0	85.002 (10)	76.221 (13)
β (°)	92.710 (6)	107.159 (11)	89.987 (10)	88.919 (11)
γ (°)	90.0	90.0	67.448 (11)	78.770 (12)
*V* (Å^3^)	1229.48(13)	2377.9(4)	623.92(13)	722.72(19)
*Z*	4	8	1	1
radiation type	Cu Kα (1.54184 Å)	Cu Kα (1.54184 Å)	Cu Kα (1.54184 Å)	Cu Kα (1.54184 Å)
μ (mm^–1^)	0.72	0.72	3.58	3.10
Data collection				
*T*_min_, *T*_max_	0.772, 1.000	0.593, 1.000	0.841, 1.000	0.841, 1.000
no. of measured, independent and observed [*I* > 2σ(*I*)] reflections	5444, 1280, 1211	24,199, 4975, 4259	7371, 2567, 2207	5684, 2928, 2346
*R*(int)	0.038	0.037	0.043	0.055
(sin θ/λ)_max_(Å^–1^)	0.630	0.631	0.631	0.628
Refinement				
*R*[*F* ^2^ > 2σ(*F* ^2^)], w*R*(*F* ^2^), *S*	0.041, 0.120, 1.10	0.050, 0.147, 1.05	0.047, 0.131, 1.05	0.054, 0.158, 1.09
no. of parameters	90	326	178	188
Δρ_max_, Δρ_min_ (e Å^–3^)	0.25, −0.17	0.55, −0.20	0.46, −0.82	0.40, −0.56

The crystal structure of L_1_ corresponded to a structure already reported in the CSD database with the CCDC number 1040607.^24^ For this reason, we decided to use the CIF file already reported for the discussion. As mentioned, L_1_, L_2_, and L_3_ have different molecular conformations, which are observed in the orientation of the triazole rings (Figure 2). In addition, Table 2 shows the values of dihedral angles involving the rotatable bonds in each case. On the other hand, the asymmetric unit of L_3_ comprises two independent molecules, of which one is a *trans* conformer, and the other shows a *cis* orientation of the triazole rings: however, with variations in their dihedral angles. From a molecular perspective, L2 is located on a crystallographic 2-fold rotation axis, which relates the two molecular halves (*Ź* = 0.5). However, L_3_ comprises two independent molecules in the asymmetric unit, each exhibiting the *trans* and *cis* conformations of the triazole rings. The supramolecular structure of L_1_ has been reported in the literature,^24^ highlighting the important role of nitrogen atoms in intermolecular interactions, involving not only the triazole rings but also the nitrogen in the pyridine moiety, which directs the crystal structure through three-dimensional connectivity. However, lacking a pyridine ring, L_2_ and L_3_ exhibit substantially altered molecular packing compared to L_1_. In L_2_, the hydrogen bonding occurs as a function of the triazole rings through C1–H1···N1 hydrogen bonds to connect molecules along the [100] direction (Table 2 and Figure 3a). This arrangement enables C–H···π (involving C5–H5 donors) that stabilize the 1D molecular chains. In this case (L_2_), the three-dimensional hydrogen bonding is broken, and the interactions between chains are mainly due to van der Waals forces (Figure 3b). In L_3_, both *cis* and *trans* configurations are present and are connected by a combination of C2B–H2B···N6A and C10A–H10A···N7B hydrogen bonds involving the triazole rings (Table 2 and Figure 3c). Despite the coexistence of these isomers in the crystal structure, each conformer is ordered in separate molecular layers that combine to form sheets stacked along the [100] direction through C1B–H1B···N7A and C11A–H11A···N1A hydrogen bonds (Table 2 and Figure 3d). From a molecular perspective, L_2_ has a 2-fold rotation axis, which is a second-order symmetry operation that relates the two molecular halves (Ź = 0.5). In contrast, neither of the conformational isomers detected in L3 exhibits a C2 symmetry, likely affected by the crystal packing and the strong hydrogen bonding.

**Table 2:** Hydrogen-Bond Geometry (Å, °) for L_2_ and L_3_ and Dihedral Angles (°) for L_1_–L_3_ ^a^

D–H···A	D–H	H···A	D···A	D–H···A
L_2_				
C1–H1···N1^i^	0.93	2.63	3.546(2)	162
C5–H5··Cg1^ii^	0.93	2.77	3.6573(11)	160
L_3_				
intra C2B–H2B···N6A	0.93	2.60	3.483(2)	159
Intra C10A–H10A···N7B	0.93	2.62	3.544(3)	172
C1B–H1B···N7A^iii^	0.93	2.59	3.512(2)	172
C11A–H11A···N1A^iv^	0.93	2.61	3.470(3)	154

Dihedral angles	
L_1_	
C4–C3–N3–C1	83.0(4)
C5–C4–C3–N3	–114.7(3)
C8–C9–N5–C11	101.9(4)
C7–C8–C9–N5	–136.0(3)
L_2_	
C4–C3–N3–C1	90.20(13)
C5–C4–C3–N3	–80.41(11)
L_3_	
C4A–C3A–N3A–C1A	94.70(16)
C5A–C4A–C3A–N3A	–121.55(16)
C8A–C9A–N5A–C11A	91.84(19)
C7A–C8A–C9A–N5A	–78.07(18)°
C4B–C3B–N3B–C1B	120.60(18)°
C12B–C4B–C3B–N3B	–29.7(2)°
C8B–C9B–N5B–C11B	92.6(2)°
C7B–C8B–C9B–N5B	–102.74(19) °

a Symmetry codes: (i) 1/2 – *x*, 1/2 – *y*, 3/2 – *z*; (ii) 1/2 + *x*, −*y*, *z*; (iii) *x*,3/2 – *y*,1/2 + *z*; (iv) *x*,–1/2 – *y*,–1/2 + *z*. Cg1: N3–N2–C2–N1–C1. Intra: interactions between both molecules in the asymmetric unit.

**Figure 3:**
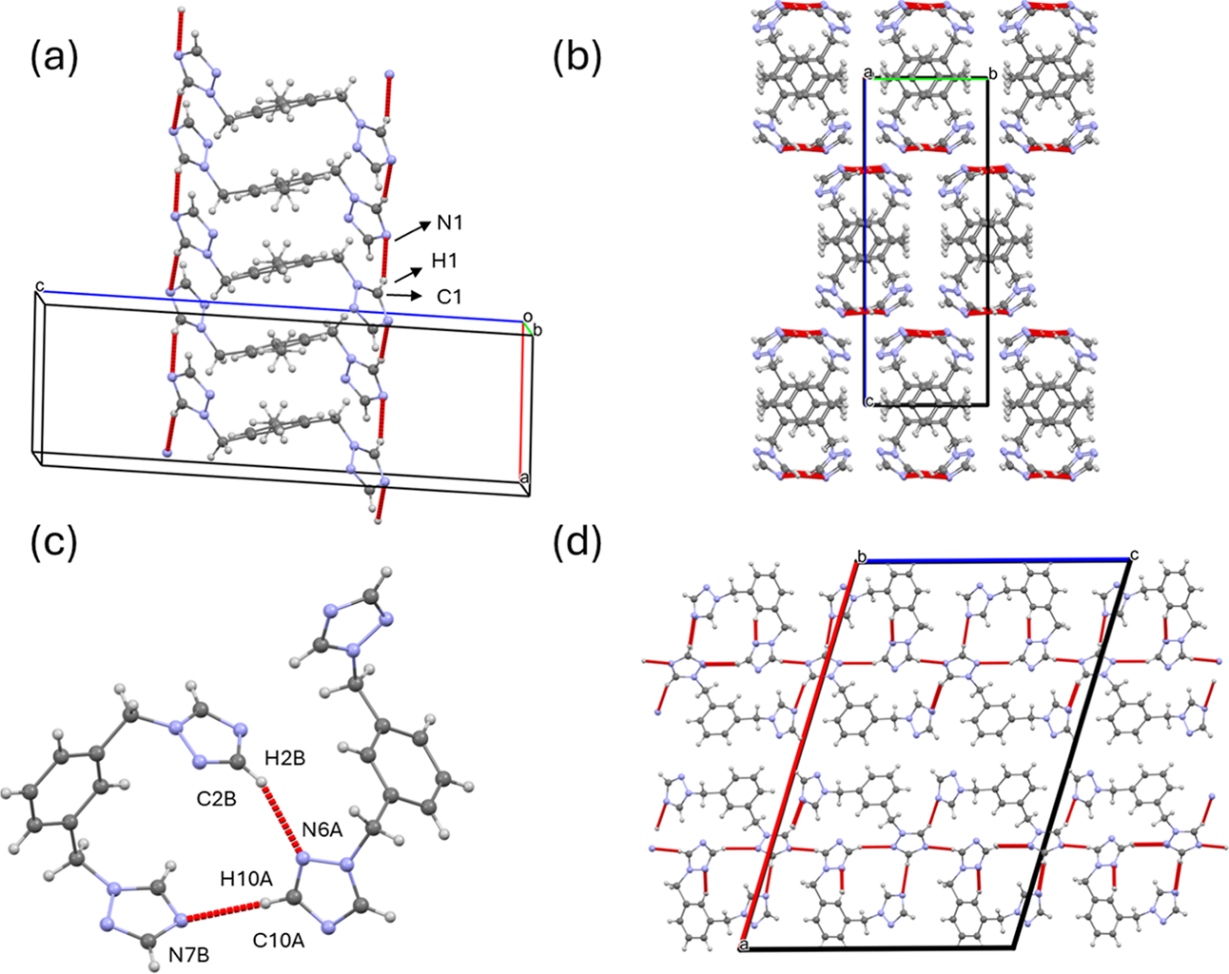
Crystal structure of L_2_ showing (a) C–H···N hydrogen bonds forming molecular chains, and (b) molecular chains viewed along [100] direction. Crystal structure of L_3_ showing (c) intermolecular C–H···N hydrogen bonds, and (d) intermolecular C–H···N hydrogen bonds in separated sheets. Red lines correspond to hydrogen bonds.

Despite the tendency of *trans* conformations of the triazole rings in L1–L3, the ligands show a different propensity in compounds C_1_–C_3_. The coordination bonds between the triazole rings and the Cu atoms (occupying centrosymmetric sites) impose structural constraints, inducing the *cis* conformation. C_1_, C_2_, and C_3_ crystallize in comparable unit cells within the *P*-1 space group (Table 1), with C_3_ already reported in the CSD database under CCDC number 764641.^25^ These similarities are a consequence of the formation of polymeric structures in all compounds, where L_1_, L_2_, and L_3_ are acting as bridging ligands, linking the Cu metal centers in these multicenter polymeric structures (Figure 4). C_2_ and C_3_ have symmetrical chains with the triazole rings oriented in similar dihedral angles to the aryl center ≈68 and 65–70°, respectively. However, in C_1_, the pyridine ring induces distortion due to a weak C10–H10A···N7 interaction (Figure 4). The polymeric chains expand along the [001] direction in all compounds, including distorted octahedral polyhedrons of copper­(II) with polyhedral volumes of 15.452, 15.971, and 15.526 Å^3^ (Table 3). The Cu–N and Cu–Cl bond lengths follow the trend: (Cu–N equatorial) C_2_ < C_3_ ≈ C_1_, (Cu–Cl axial) C_2_ > C_3_ > C_1_, which suggests stronger Jahn–Teller distortion in C_2_ with minor effect in C_1_. This phenomenon is also observed in the quadratic elongation values 1.048, 1.072, and 1.052 for C_1_, C_2_, and C_3_, respectively. In the supramolecular architectures, chlorine atoms link the chains. In C_1_, essentially C–H···Cl and C–H···π hydrogen bonds build the 3-dimensional connectivity, while in C_2_ and C_3,_ C–H···N interactions also participate in the crystal formation, similarly to the previously observed for C_3_ (Table 4).^25^ Polymeric structures in all cases are formed due to the coordination of the 1,3-bis­(triazolyl) ligand. Despite the tendency to form similar chains, differences are observed in the dihedral angles between the 1,3-bis­(triazolyl) rings and the plane containing the axial N atoms, which are 26.35(5)°/76.26(5)°, 58.19(6)°/73.48(6)°, and 45.16(6)°/75.52(6)° for C_1_, C_2_, and C_3_, respectively.

**Figure 4:**
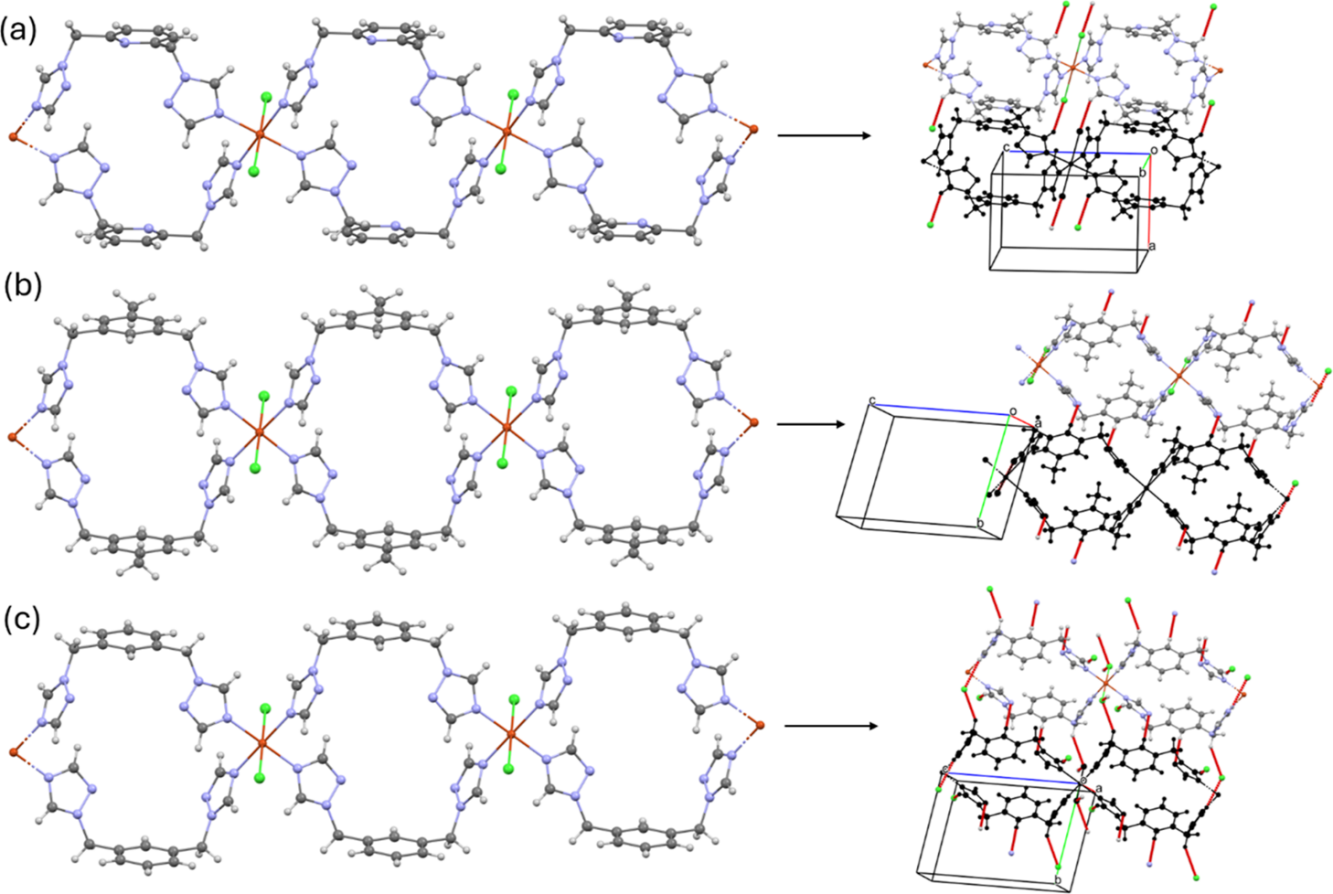
Polymeric structures of compounds (a) C_1_, (b) C_2_, and (c) C_3_, showing the C–H···Cl and C–H···N hydrogen bonds that connect neighboring chains. Red lines correspond to hydrogen bonds.

**Table 3:** Cu–N and Cu–Cl Bond Distances (Å) and Angles (°) for C_1_, C_2_, and C_3_

	Bond Distance		
	C_1_	C_2_	C_3_
Cu–N(5)	2.052(2)	2.006(2)	2.0212(16)
Cu–N(1)	2.026(3)	2.024(3)	2.0454(17)
Cu–Cl	2.7896(9)	2.9521(8)	2.8194(8)
	Bond Angles		
Cl(1)–Cu(1)–N(1)	90.70(8)	91.50(12)	91.95(6)
Cl(1)–Cu(1)–N(5)	88.59(8)	88.90(12)	88.61(6)
N(1)–Cu(1)–N(5)	92.09(10)	91.18(12)	90.55(7)
	87.91(10)	88.83(12)	89.45(7)

**Table 4:** Hydrogen-Bond Geometry (Å, °) for C_1_, C_2_, and C_3_ ^a^

D–H···A	D–H	H···A	D···A	D–H···A
C_1_				
C3–H3A···Cl1^i^	0.97	2.75	3.692(3)	164
Intra C2 –H2···Cl1	0.93	2.74	3.340(3)	123
C10–H10B···Cl1^ii^	0.97	2.77	3.661(3)	153
C12–H12···Cl1^ii^	0.93	2.64	3.445(4)	145
C5–H5···Cg2^iii^	0.93	2.75	3.448(4)	133
C11–H11···Cg1^v^	0.93	2.90	3.494(4)	123
C_2_				
C9–H9···N6^vi^	0.93	2.47	3.402(5)	180
Intra C2–H2···Cl1	0.93	2.79	3.412(4)	125
C13–H13B···Cg4^vii^	0.96	2.70	3.578(5)	152
C_3_				
C3–H3A···Cl1^viii^	0.97	2.77	3.552(3)	138
Intra C2 –H2···Cl1	0.93	2.68	3.302(3)	125
C9–H9···N6^ix^	0.93	2.58	3.497(3)	171
C11–H11···Cl1^x^	0.93	2.68	3.542(3)	155

a Symmetry codes: (i) *x*,1 + *y*,*z*; (ii) 1 – *x*,1 – *y*,2 – *z*; (iii) −*x*,2 – *y*,2 – *z*; (iv) −*x*,2 – *y*,1 – *z*; (v) −*x*,1 – *y*,2 – *z*; (vi) 1 – *x*,–y,–1 – *z*; (vii) −*x*,1 – *y*,–1 – *z*; (viii); (viii) *x*,1 + *y*,*z*; (ix) −*x*,1 – *y*,1 – *z*; (x) 1 – *x*,–*y*,1 – *z*. Cg1: N1–N2–C1–N3–C2, Cg2: N4–N6–C11–N5–C12, Cg4: C4–C5–C6–C7–C8–C9. Intra: interactions between both molecules in the asymmetric unit.

From the described results, compounds that share structural features with C_3_, particularly in terms of conformational freedom and geometry of the bis-triazole spacers and properties of auxiliary anions, tend to form preferentially one-dimensional coordination polymers, as observed in the case of C_1_ and C_2_. In C_3_, the 1,3-bis­(triazolyl) arrangement imposes specific bite angles and a semirigid bridging mode that restricts the growth of the coordination framework to double-chain 1D motifs.^25^ This behavior is consistent with other bis-triazole systems featuring comparable spacer orientations, where the connectivity enforced by the 1,3-linkage confines the propagation of the polymer to a single crystallographic direction.^25^ In contrast, altering the spacer geometry–such as employing 1,2- or 1,4-bis­(triazolylmethyl)­benzene–enables more favorable angular dispositions for planar or multidirectional metal bridging, thus giving rise to 2D (4,4) networks, as illustrated within the same ligand series.^25^ Additional evidence from flexible aliphatic bis-triazole frameworks demonstrates that even subtle variations in spacer length or conformational freedom can promote dimensional transitions from 1D chains to 2D sheets or full 3D architectures.^26^ Likewise, systems incorporating highly connected auxiliary anions, particularly Keggin-type polyoxometalates, frequently exhibit enhanced dimensionality due to the polyanion’s ability to act as a multidentate connector, generating 2D or 3D metal–ligand–anion frameworks.^27,28^ Furthermore, changes in ligand-to-metal stoichiometry have also been shown to modulate the resulting topology, reinforcing the idea that small structural or synthetic modifications can drive pronounced shifts in dimensionality.^27^ Collectively, these studies indicate that the intrinsic geometric constraints of the ligand backbone–modulated by the coordinating anion–dictate whether Cu­(II) bis-triazole complexes assemble as 1D chains or evolve into higher-dimensional topologies, underscoring the sensitivity of these systems to spacer orientation, flexibility, and auxiliary components.

### 2.3 Computational Calculations

Figure 5 presents the formation energy (Δ*E*_f_) profiles for the polymers, quantifying the incremental energy required to build each chain monomer-by-monomer up to the experimental X-ray structure. The bridging compounds L_1_, L_2_, and L_3_ exhibit highly linear behavior, as expected given that geometry optimization was not performed, and all ligand and complex fragments retained their crystallographic geometries. The slopes represent the amount of energy involved when a new monomer is added to each type of chain: C_1_ = −1.84, C_2_ = −2.97, and C_3_ = −2.47 kcal/mol, indicating that the complexes gain electronic stability through the chain growth, with C_2_ standing as the one with the best stabilization.

**Figure 5:**
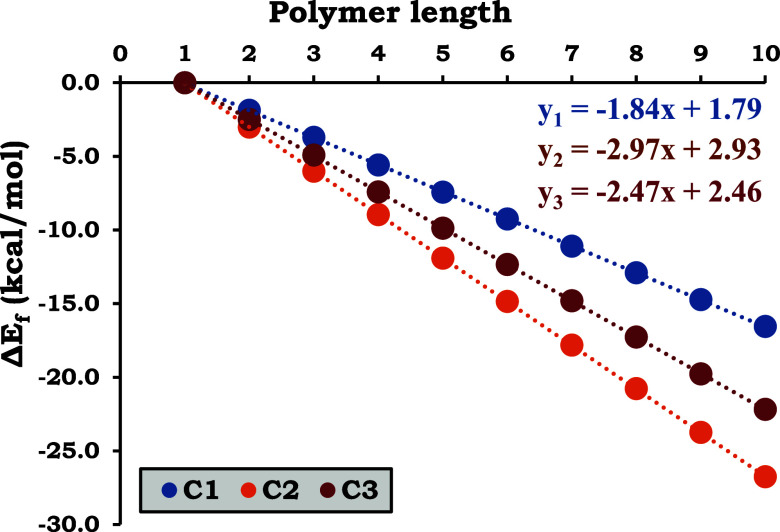
Calculated polymer formation energy (Δ*E*_f_) for chains up to 10 Cu atoms in the three copper polymers (R_1_ = R_2_ = R_3_ = 0.9999).

Figure 6 shows the results for the Δ*E*_avg_ for the complex chains, which represents the stabilization energy per monomer relative to its uncoordinated state. These exhibit only negative energies that decrease over the number of units until a stabilization is reached near the 10 monomers with the values: C_1_ = −1.65, C_2_ = −2.68, and C_3_ = −2.22 (kcal/mol). These are approximately the amount of Δ*E*_avg_ expected for longer chain polymers, as the variation in this energy when increasing the number of monomers decreases dramatically, as presented in Figure 6 as crosses that reach ΔΔ*E*_avg_ values between −0.02 and −0.04 kcal/mol at 9 to 10 units quickly.

**Figure 6:**
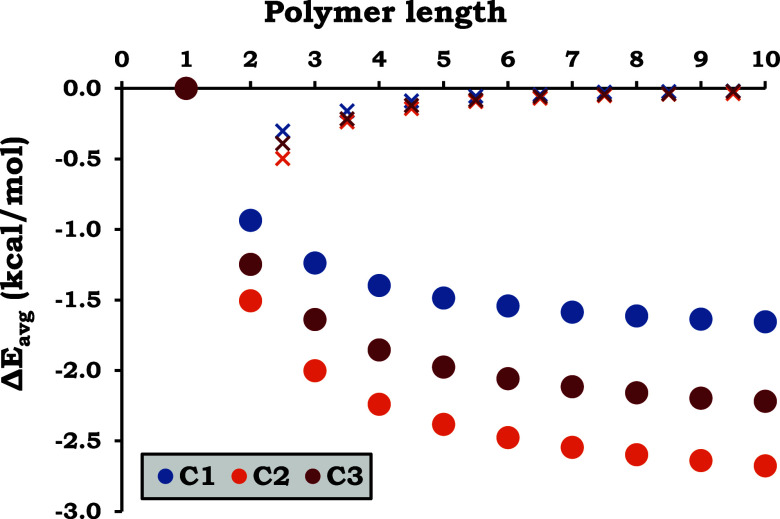
Average stabilization energy per monomer (Δ*E*_avg_) for the three copper polymers. The ΔΔ*E*_avg_ between two chain lengths is indicated as crosses placed within the two respective chain numbers.

## 3 Conclusion

Two copper coordination polymers were obtained using the ligands 2,6-bis­((1*H*-1,2,4-triazol-1-yl)­methyl)­pyridine and 3,5-bis­(1,2,4-triazol-1-ylmethyl)­toluene, in which the metal centers adopt octahedral geometries. The polymers were isolated as crystalline solids in good yields and are stable in air.

GFN2-xTB calculations confirm chain stabilization upon polymerization for all three polymers. Chains of 1–10 monomers yield negative Δ*E*_f_ (highly linear) and Δ*E*_avg_ values that converge by n = 10. In this computational modeling, L_2_ stands out as the most stabilizing ligand when forming polymers with Cu­(II) ions, with a Δ*E*_f_ slope of −2.97 kcal/mol and a reached Δ*E*_avg_ limit of −2.68 kcal/mol. Crystallographic studies reveal that central ring variations in L_1_ (pyridine), L_2_, and L_3_ alter ligand conformations, point-group symmetries, and *cis*/*trans* isomer distributions. Nevertheless, C_1_, C_2_, and C_3_ consistently form analogous 1D polymeric chains, consistent with literature precedents and computational calculations.

## 4 Experimental Section

### 4.1 General

All chemicals were commercially available, and the reagent-grade solvents were dried and distilled before use. Melting points were determined on a Mel-Temp 1101D apparatus and are reported uncorrected. Elemental analysis (C, H, and N) was performed with a Thermo Scientific FLASH 2000 CHNS/O Analyzer. Fourier transform infrared (FTIR) spectra were recorded using an ATR module on a Thermo Nicolet NEXUS FTIR spectrophotometer. The mass spectrum was recorded on a SHIMADZU-GCMS 2010-DI-2010 spectrometer (Scientific Instruments Inc., Columbia, WA, USA) equipped with a direct inlet probe operating at 70 eV (positive mode). The ligands 2,6-bis­((1*H*-1,2,4-triazol-1-yl)­methyl)­pyridine (L_1_), 3,5-bis­(1,2,4-triazol-1-ylmethyl)­toluene (L_2_) and 2,6-bis­((1*H*-1,2,4-triazol-1-yl)­methyl)­benzene (L_3_) were prepared according to literature methods.^29–31^

### 4.2 Synthesis of Coordination Polymer 2,6-bis­((1*H*-1,2,4-Triazol-1-yl)­methyl)­pyridine-CuCl_2_ (C_1_)

1.19 mmol of CuCl_2_ was dissolved in 5 mL of methanol (MeOH) in a 50 mL flask at room temperature and added to 1.08 mmol of L_1_, previously dissolved in 10 mL of MeOH, slowly. The resulting solution was kept under constant agitation for 6 h. Subsequently, the former solid was filtered, washed with MeOH, dichloromethane and ethyl ether, and dried under vacuum. Yield: 83%; mp: 250 °C. Anal. Calcd. for C_11_H_11_Cl_2_N_7_Cu: C, 35.17; H, 2.95; N, 26.10. Found: C, 35.16; H, 2.90; N, 26.07. ESI mass spectrum *m*/*z*: 304.0355 [M^+^ – 2Cl]. IR (ATR cm^–1^): 1531, 1427, 1280 cm^–1^.

### 4.3 Synthesis of Coordination Polymer 3,5-bis­(1,2,4-Triazol-1-ylmethyl)­toluene-CuCl_2_ (C_2_)

In a 50 mL flask, 0.39 mmol of L_2_ was dissolved in 10 mL of MeOH at room temperature, and 0.43 mmol of CuCl_2_ in 2 mL MeOH was added. The mixture was kept under constant agitation for 6 h. The formed solid was filtered, washed with MeOH, dichloromethane, and ethyl ether, and dried under vacuum. Yield: 79%; mp 283 °C. Anal. Calc. for C_13_H_14_Cl_2_N_6_Cu: C, 40.17; H, 3.63; N, 21.62. Found: C, 40.12; H, 3.58; N, 21.79. ESI mass spectrum: *m*/*z* 317.0567 [M^+^ – 2Cl]. IR (ATR cm^–1^): 1527, 1435, 1280 cm^–1^.

### 4.4 X-ray Diffraction Analysis

The X-ray diffraction data were acquired at room temperature using Cu Kα (λ = 1.54184 Å) radiation. For measurements, an Agilent SuperNova, Dual, Cu at Zero, Atlas four-circle diffractometer equipped with a CCD plate detector was used, through ω scans. The collected frames were integrated and corrected for the absorption effect using the CrysAlis PRO software package (CrysAlisPro 1.171.39.46e, Rigaku Oxford Diffraction, 2018). The crystal structure was solved using an iterative algorithm^32^ and completed by a difference Fourier map. The crystal structures were refined by using the SHELXL2018/3 program.^33^ Molecular and supramolecular graphics were carried out using the software Mercury.^34^ The Crystallographic Information File (CIF files) has been deposited in the Cambridge Crystallographic Data Center (CCDC) with deposition numbers: CCDC-2473469, 2473468, 2473467, and 2473470 for compounds L_2_, L_3_, C_1_, and C_2_, respectively.

### 4.5 Computational Calculations

A series of computational calculations was carried out using the GFN-2/xTB method.^35^ This semiempirical extended tight binding method has been shown to retrieve excellent results with great cost-benefit balance in many applications, such as the modeling of biological systems and the growth of polymer chains.^36–38^ Single-point calculations were performed to assess the energetic stability of the polymerization mechanism involving these metal complexes. For this purpose, hydrogen atoms were introduced into the crystal structure at standard distances. The gas-phase computational approach was selected to accurately represent the solvent-independent nature of the polymerization process observed in the experimental work. To model sequential monomer binding energetics, model chains of 1–10 units were constructed for each polymer, replicating their crystallographic geometries. Δ*E*_f_ was computed via single-point GFN2-xTB calculations (eq 1), capturing raw binding energies per polymerization step without geometry optimization of complexes or ligands.

1 $${\Delta E}_{f}=E_{chain,N}+{\left(2N-2\right)E}_{ligand}-{\left(N\right)E}_{monomer}$$

Where Δ*E*_f_ is the delta energy of polymer formation, *E*_chain,N_: polymer chain energy, *E*_ligand_: ligand energy, *E*_monomer_: energy of an isolated monomer, and N: number of monomer units. As an example, the polymer formation reaction for the first dimer of compound L_1_ is shown in Figure 7. As seen, the formation reaction occurs between *N* monomer units, releasing (2*N* – 2) ligands to the medium. Similarly, the average stabilization energy per monomer (Δ*E*_avg_) is a measurement of how much each monomer is being stabilized in the chain formation compared to its uncoordinated state. It was calculated according to eq 2 as

2 $$\Delta E_{avg}=\frac{{\Delta E}_{f}}{N}=\frac{E_{chain,N}+{\left(2N-2\right)E}_{ligand}}{N}-E_{monomer}$$

**Figure 7:**
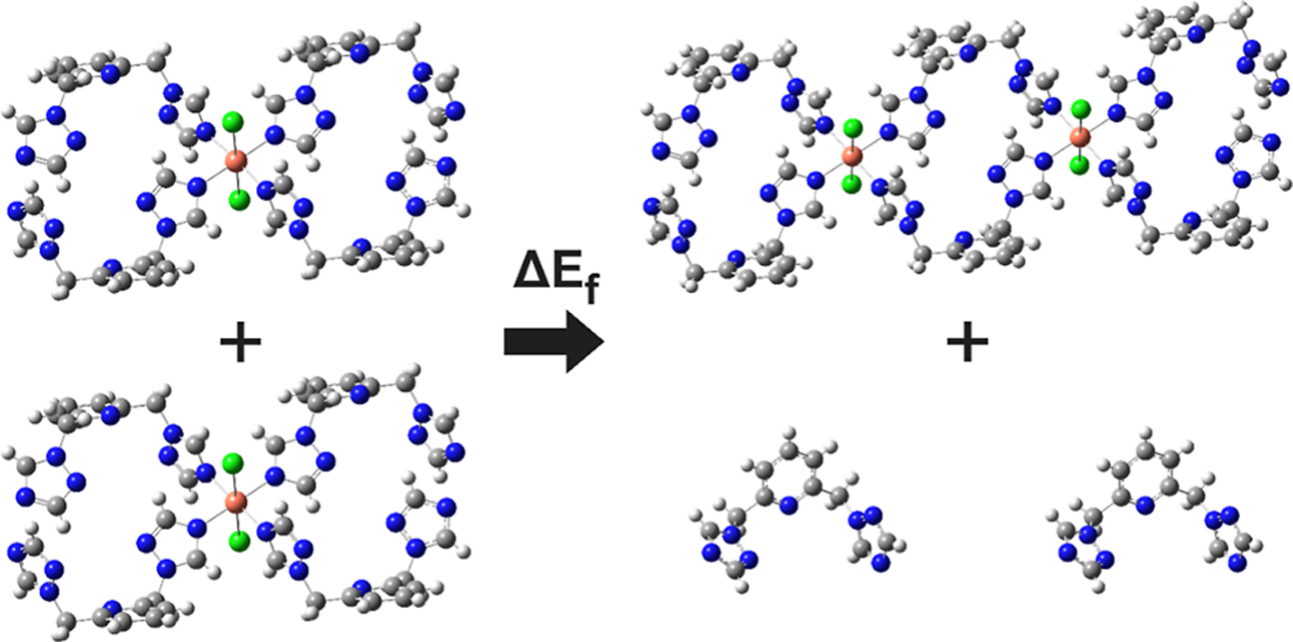
Chemical reaction for the delta energy of polymer formation (Δ*E*_f_) for the dimer formation of compound L_1_: 2L1_2_Cu → L1_6_Cu_2_ + 2L1.

## Supplementary Material



## Notes

Supporting Information associated with this article includes: Figure S1. FT-IR spectra of L_1_ Figure S2. FT-IR spectra of L_2_ Figure S3. FT-IR spectra of C_1_ Figure S4. FT-IR spectra of C_2_ Figure S5. Mass spectrum of C_1_ Figure S6. Mass spectrum of C_2_ (PDF)
