# Supplementary material for: New Coordination Polymers with Unexpected Octahedral copper(II) Geometry: Synthesis, Supramolecular and Theoretical Study
Source: ACS Omega. 2026 May 11;11(20):29390–8. doi: 10.1021/acsomega.5c07364 (PMC13216925; doi:10.1021/acsomega.5c07364)
Supplement: Supplementary file 1 [file ao5c07364_si_001.pdf]

New coordination polymers with unexpected octahedral copper(II) geometry: synthesis, supramolecular and theoretical study

Yair Alvarez-Ricardo[1], Nicolás Puentes-Díaz[2], Mario A. Macías[3], Jorge Alí-Torres[*2], John Hurtado*[*1]*

[1]Grupo de investigación en Química Inorgánica, Catálisis y Bioinorgánica, Departamento de Química, Universidad de los Andes, Carrera 1 No. 18A-12, 111711, Bogotá-Colombia. [2]Departamento de Química, Universidad Nacional de Colombia – Sede Bogotá, Bogotá, Colombia. [3]Cristalografía y Química de Materiales, CrisQuimMat, Departamento de Química, Universidad de los Andes, 111711, Bogotá-Colombia. * jj.hurtado@uniandes.edu.co; Tel.: +57-1-3394949 (ext. 3468), ORCID: https://orcid.org/0000-0002-0511-9719. * jialit@unal.edu.co, Tel.: +57-601-3165000 (ext. 10608), https://orcid.org/0000-0003-1354-8713.

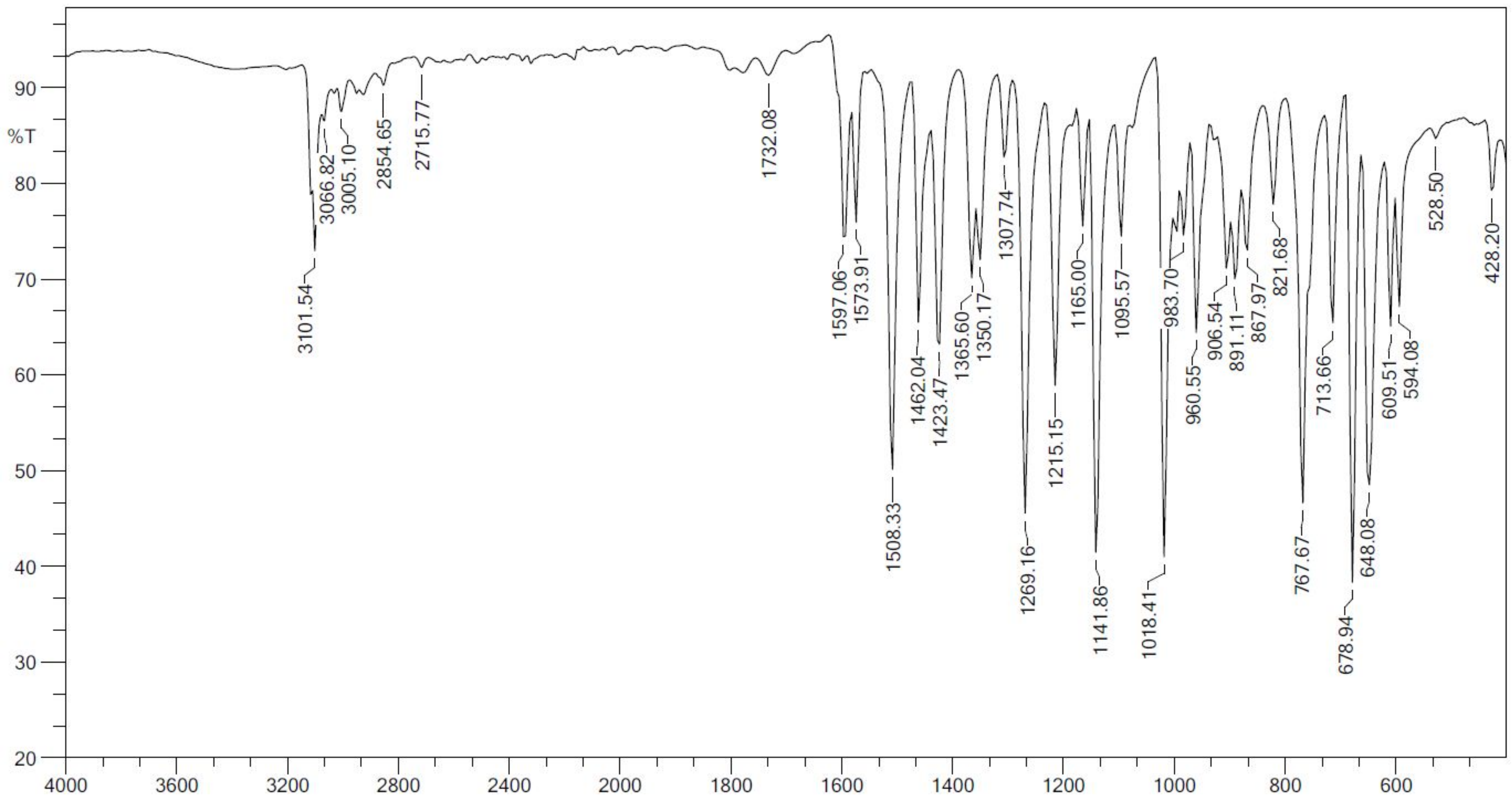

Figure S1. FT-IR spectra of **L**$_1$ using an ATR module.

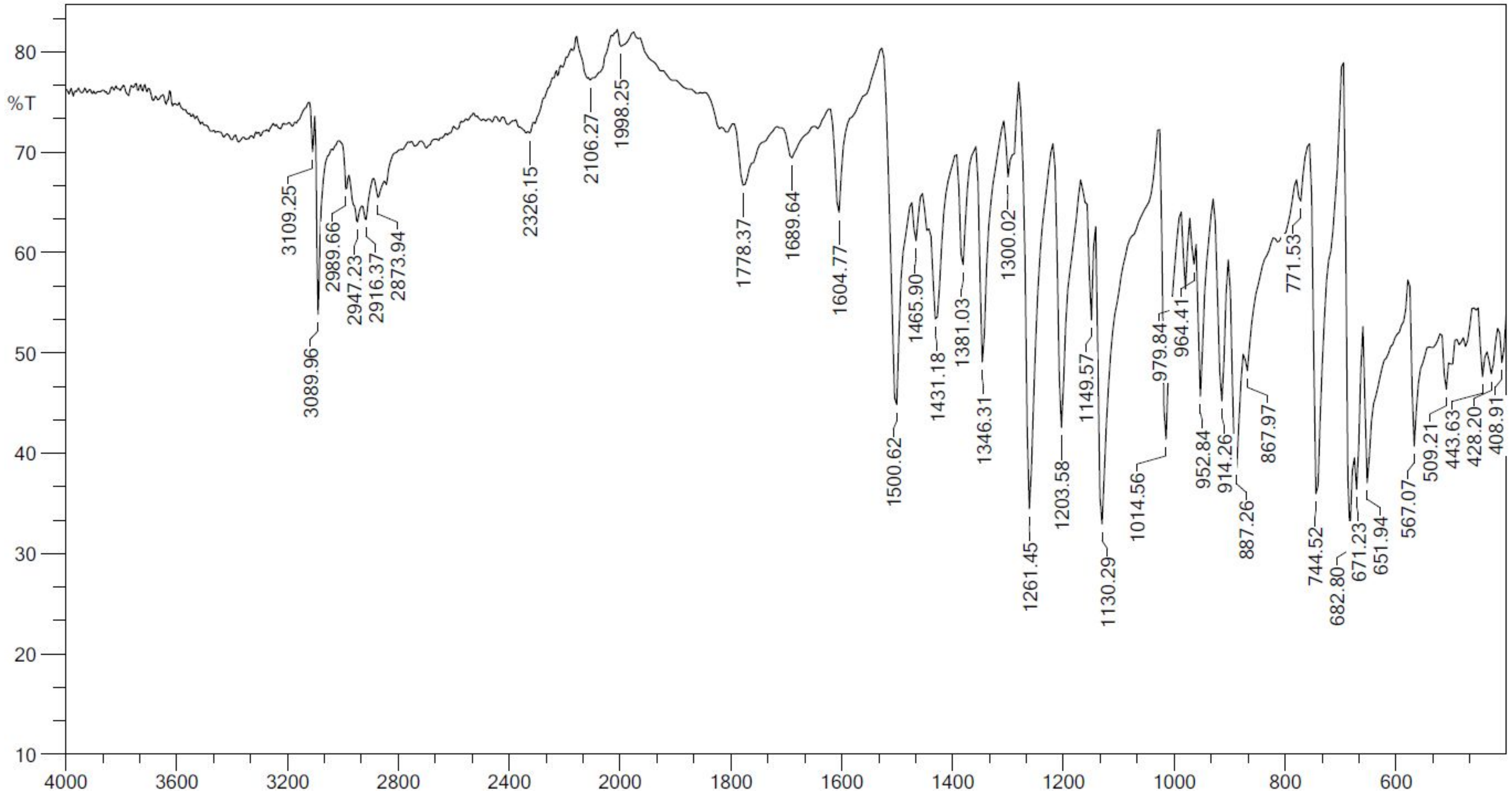

Figure S2. FT-IR spectra of L_2 using an ATR module.

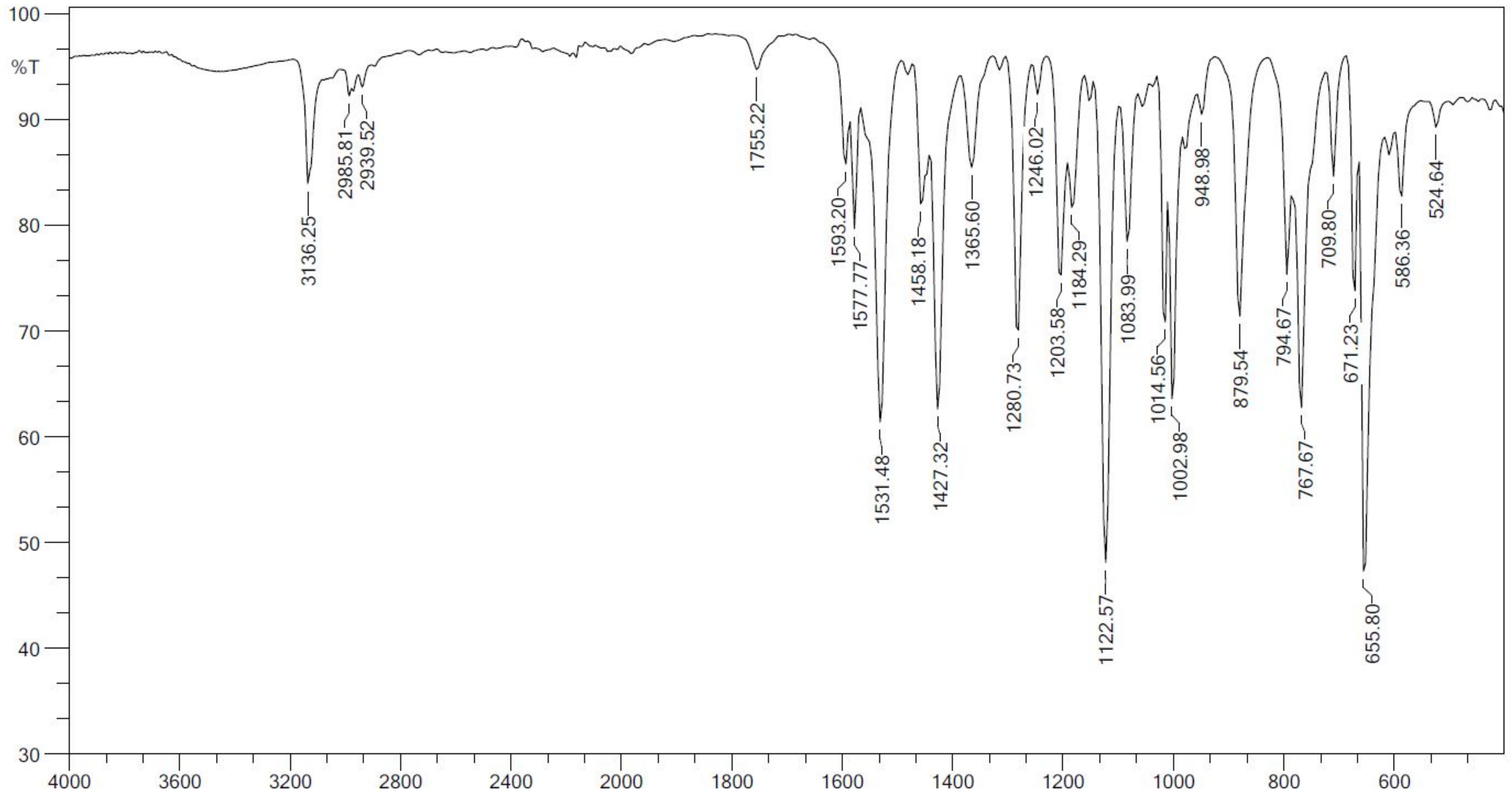

Figure S3. FT-IR spectra of $\mathbf{C_1}$ using an ATR module.

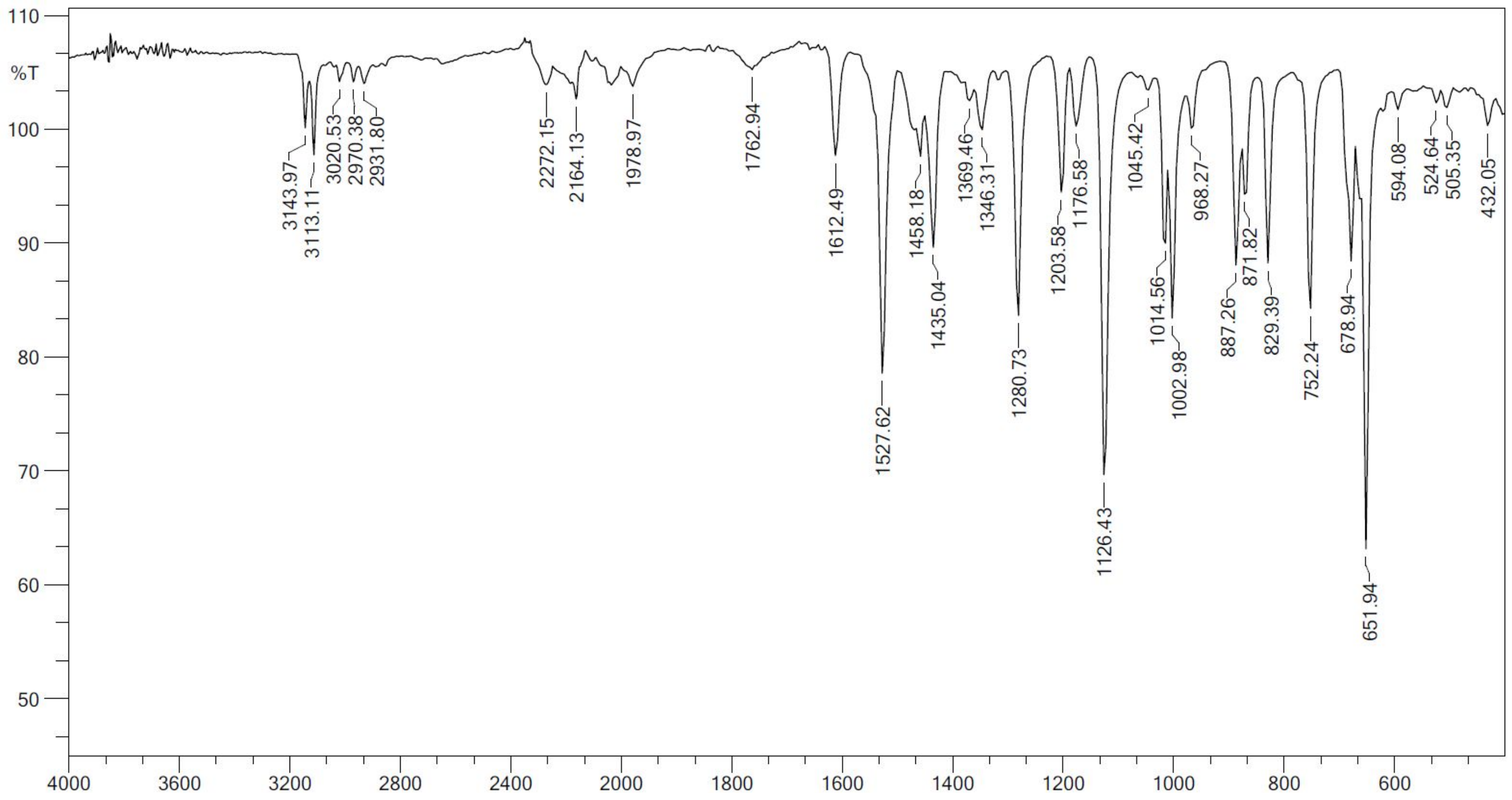

Figure S4. FT-IR spectra of $\mathbf{C_2}$ using an ATR module.

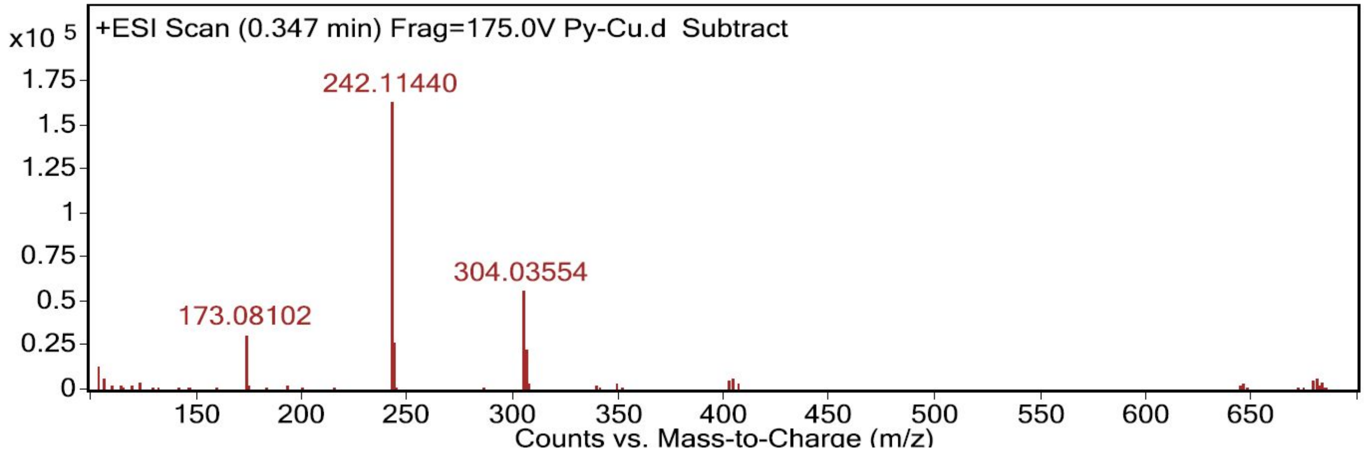

Figure S5. Mass spectrum of $\mathbf{C_1}$

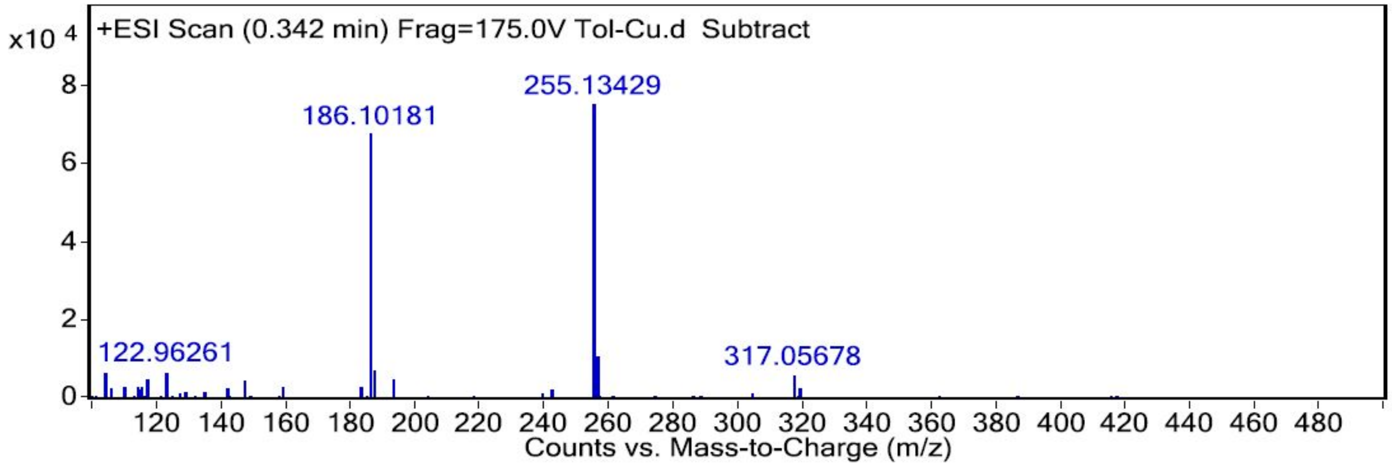

Figure S6. Mass spectrum of $\mathbf{C_2}$
